# Utilization of Bone Alkaline Phosphatase (BAP) and Tartrate Resistant Acid Phosphatase (TRAP) as Biomarkers of Eggshell Quality and Bone Metabolism in Broiler Breeders and Progeny

**DOI:** 10.1111/jpn.14075

**Published:** 2024-12-01

**Authors:** A. D. Magnuson, N. Boonsinchai, J. Caldas, J. England, C. Coon

**Affiliations:** ^1^ Center of Excellence for Poultry Science University of Arkansas Fayetteville Arkansas USA; ^2^ CP Group Bangkok Thailand; ^3^ Aviagen Incorporated Huntsville Alabama USA

**Keywords:** biomarkers, bone strength, broiler breeder, eggshell quality, progeny

## Abstract

Eggshell breakage and broiler bone disorders are major problems for the breeder and broiler industries which are linked to mineral metabolism and animal genetics. The purpose of this work was to discover the link between individual animal phenotypic differences in mineral metabolism against concentrations of novel plasma biomarkers including tartrate resistant acid phosphatase (TRAP) and bone alkaline phosphatase (BAP). A subset of hens were selected from a flock of Cobb 500 breeders with the best or worst eggshell quality based upon dual energy x‐ray absorptiometry (DEXA) and specific gravity (SG). Breeders were defined as having good eggshell quality (SG ≥ 1.080), or poor eggshell quality (SG < 1.080). Progeny hatched from breeders with good or poor eggshell quality were reared to 2 week of age and blood and bone samples were obtained after euthanasia. In both breeders and progeny, plasma concentrations of BAP and TRAP were measured, and bone mineral density was evaluated by DEXA. Results showed that breeders selected for eggshell quality had significantly different plasma concentrations of BAP (Good = 326.5 pg/mL, Poor = 253.2 pg/mL), and TRAP activity (Good = 2203 U, Poor = 4985 U). Breeders selected for eggshell quality produced progeny with different bone breaking strength (Good = 1.61 kg/mm, Poor = 1.47 kg/mm), tibia ash (Good = 45.9%, Poor = 42.2%), plasma BAP (Good = 372.3 pg/mL, Poor = 312.4 pg/mL), and lower plasma TRAP activity (Good = 18010 U, Poor = 23590 U). These data suggest that there is a strong correlation between the eggshell quality of breeders, performance and bone strength of progeny, and plasma of concentrations of BAP and TRAP in both breeder hens and progeny.

**Abbreviations**
BAP: bone alkaline phosphatase
DEXA: dual energy x‐ray absorptiometry
EP: egg production
EW: egg weight
HDEP: hen‐d EP %
HHEP: hen‐housed EP %
SG: specific gravity
sPLSDA: sparse partial‐least squares discriminant analysis
SWUSA: shell weight per unit surface area
TRAP: tartrate resistant acid phosphatase

## 1 Introduction

Improving broiler breeder performance to produce high quality eggs with healthy rapidly growing progeny is a major focus of the broiler industry (Bain 2005; Peebles and Brake 1987; Roque and Soares 1994). Breeders and progeny are susceptible to mineral metabolism related disorders which cause poor bone health and negatively affect performance (Tůmová, Gous, and Tyler 2014; Waldenstedt 2006). The limiting macro‐mineral for eggshell and bone synthesis is calcium (Ca) which is impacted by its ratio with phosphorus (P), both of which are supplied through both dietary intake and medullary bone resorption (Manangi, Maharjan, and Coon 2018; Salisbury, Cowieson, and Gous 2021). Eggshell thickness is a parameter selected for in breeders as it can dictate hatchability of progeny and correlates with eggshell breakage (Aalaei et al. 2018; Bennett 1992). Many factors modulate the eggshell quality of breeders including stress, genetics, disease, nutrition, and environmental conditions (Ketta and Tůmová 2016). Improper dietary Ca:P balance can rapidly impact eggshell quality, however previous work has suggested there are individual phenotypic differences amongst breeders (Ekmay et al. 2012). Thus, there is potential to select breeders for high eggshell quality through related plasma circulating Ca and P metabolism biomarkers.

Several hormones regulate Ca metabolism in breeders such as parathyroid hormone, calcitriol, calcitonin, and fibroblast growth factor 23 (Magnuson 2015). These signalling molecules maintain the blood concentration of Ca through several organs including the kidney, small intestine, and bone (Proszkowiec‐Weglarz and Angel 2013). Apart from dietary intake, medullary bone resorption provides a consistent and on demand supply of Ca for eggshell Ca‐carbonate synthesis (Kerschnitzki et al. 2014). The two major types of cells responsible for bone mineral turnover include osteoblasts and osteoclasts which secrete bone alkaline phosphatase (BAP) and tartrate resistant acid phosphatase (TRAP), respectively (Chao, Wu, and Janckila 2010; Sharma, Pal, and Prasad 2014). BAP is not localised in the bone as it can permeate into the circulation where it is transported with other molecules from osteocytes. Historically BAP has been utilized as an indicator of bone formation in various models including murine and human, however, BAP has had limited application in poultry including breeders (Brichacek and Brown 2019). Likewise, TRAP has been seldom used in breeder research despite being correlated with bone resorption and demineralisation (Ekmay et al. 2012). Genetics influences the secretion of these enzymes, however, it has yet to be investigated how concentrations of BAP and TRAP correlate with eggshell quality of breeders and bone strength and growth rate of progeny.

The first objective of this study was to determine if there is a correlation between eggshell quality, bone health, and plasma concentrations of BAP and TRAP in breeders. The second objective was to elucidate the relationship of these enzymes and heritability between progeny and parent stock. The hypothesis was that plasma BAP and TRAP would be correlated to eggshell thickness and bone density and that these parameters would be heritable between parent stock and progeny.

## 2 Methods

### 2.1 Animals and Handling

A flock of 850 Cobb 500 hens was delivered to the production house at the age of 20 weeks. Each hen was assigned an identification number and individually caged (47 cm high, 30.5 cm wide, 47 cm deep) with one feeder and nipple water drinker per cage. Hens were offered Cobb feed daily (Table S1) utilizing a Cobb breeder feed allocation programme (Table S2) (Cobb‐Vantress 2005). Daily allotted feed intake was controlled during rearing and was increased every 8% increase in egg production (EP) beginning from 5% production to peak. Birds were maintained in environmentally controlled conditions with temperature at 22°C and humidity at 40%. Lighting schedule began with 12 h per day when pullets were 21 week of age and increased 1 h per week for the next 2 weeks until reaching its apex at 14 h per day. Light duration was further increased to 15 and 16 h per day at 20%, and 50% EP, respectively.

### 2.2 Breeder Selection & Sampling

Breeder EP was recorded daily, and egg weight (EW) recorded 2 days a week. Eggs which were soft shelled, cracked, dirty, or double yolk were recorded as such. Beginning at 30 weeks of age, the eggs weighed for each hen were analyzed by dual energy x‐ray absorptiometry (DEXA) (GE ®Lunar Prodigy) for 16 weeks to determine eggshell mineral concentration and thickness. Regression equations developed for determining eggshell parameters from the DEXA scanning (England et al. 2012) were used on eggs from the flock of 850 hens to select 20 hens which produced eggs with the poorest eggshell quality (Specific gravity (SG) < 1.080) and 20 hens which produced eggs with the best eggshell quality (SG ≥ 1.080). Each individually caged hen was considered a replicate with a total of 20 replicates per eggshell quality group. The SG of each egg was measured through immersion in salt solutions ranging in SG from 1.060 to 1.095 with a concentration gradient of 0.005 between solutions. After hens were selected for poor or good eggshell quality, the breeder hens were subjected to whole body scanning with DEXA to determine bone quality based upon total body mineral content. Previously developed equations for DEXA to determine whole body composition of hens using DEXA were used (Salas et al. 2019).

At the end of the 16 week study, blood was drawn from the wing vein of breeders selected for good or poor eggshell quality. Blood was collected within 30 min of an egg being laid to account for oviposition. Previous work by Ekmay et al. (2012) demonstrated that breeder plasma BAP and TRAP are influenced by lighting, feeding time, and oviposition, thus we focused on oviposition as the parameter to conserve for normalizing plasma BAP and TRAP as most eggs were laid 20–24 h post feeding (Ekmay et al. 2012). Blood samples were stored in heparinized blood collection tubes on ice until being centrifuged using the methodology described by Ekmay et al. (2012) for plasma separation, and stored at −20°C until analysis for TRAP and chicken specific BAP (Ekmay et al. 2012). Hens were then euthanized through CO_2_ gas asphyxiation and right tibias removed for the measurement of breaking strength and bone ash concentration.

Plasma chicken specific BAP was analysed using a quantitative competitive immunoassay test kit (Neobiolab, USA) developed following cGMP procedures including testing for precision (Intravariability CV: 4.2%–5.9%, Intervariability CV: 7.6%–9.8%), dilutional linearity (84%–99%), and recovery (83%–96%) of the chicken‐BAP protein (Andreasson et al. 2015). Notably Neobiolab couldn't verify if the chicken‐BAP ELISA kit detected exclusively the activated form or total amount of this protein. While not provided by the vendor, this work determined a limit of detection at 8.79 pg/mL and a limit of quantitation at 26.37 pg/mL for the BAP ELISA kit.

Plasma TRAP was analysed using a micro‐titre plate spectrophotometer absorptiometry technique described by Lau et al. (1987). Tibias were analysed for bone‐breaking force by the sheer force measurement method described by Wilson (1991), utilizing an Instron Universal Testing Machine (Model 1123, Instron Corp., Canton, MA) (Wilson 1991). Following the sheer test, the tibias were defatted in a container of 180 proof alcohol for 24 h followed by refluxing in petroleum ether in a Soxhlet apparatus for 48 h. The defatted tibia samples were oven‐dried at 110°C for 24 h and ashed in ceramic crucibles for 24 h at 600°C. Ash content was determined as dry, fat‐free tibia, and expressed as grams of ash/bone and as a percentage of the defatted tibia weight.

### 2.3 Progeny Generation & Sampling

Hens which were selected for poor or good eggshell quality were artificially inseminated twice a week starting at week 46 through week 50. Semen used was collected and combined from forty 28 week of age Cobb MX broiler breeder males through abdominal massage method (Cerrate et al. 2019). Collected semen was pooled and sperm cell concentration was measured using an IM Micro‐reader using an optical density of 381 nm (King, Holsberger, and Donoghue 2000). Lake solution was used to dilute semen to a concentration of 2 million cells/50 µL for a consistent concentration of sperm utilized for insemination. Following artificial insemination, eggs were collected for a 6‐day duration from the selected hens and stored in an egg cooler until they were placed for incubation and hatching. Three eggs per hen were hatched with chicks from each hen being considered a replicate for a total of 20 replicates per treatment group. Live hatched chicks from either the good or poor eggshell quality hens were given individual wing bands and reared in separate floor pens. Chick mortality was measured daily along with nipple water drinker adjustments. Chicks were offered a starter diet (Table S3) for 14 days and then blood was drawn from the external jugular vein. Chicks were then euthanized through CO_2_ asphyxiation and right tibias removed for measuring breaking strength and bone ash. Chicks were scanned before tibia removal for the determination of whole body composition using the DEXA with equations developed by Caldas et al. (2018). Plasma from the chicks was also subjected to analysis for both TRAP and chick specific BAP using the same methodology as used with the parent stock.

### 2.4 Statistical Analysis

All data were analysed by using one‐way ANOVA (version 9, SAS Institute, 1989, Cary, NC). All statements of significance are based on testing at *p* ≤ 0.05. Differences between treatments were explored using sparse partial least squares discriminant analysis (sPLSDA) and heatmapping through MetaboAnalyst software with data normalised using Pareto scaling and a false discovery rate (Chong et al. 2018). Utilization of sPLSDA was meant to provide visual aid for parameters responsible for treatment separation between hens selected for eggshell quality.

## 3 Results

### 3.1 Egg & Production Parameters

No statistical differences were found between hens selected for eggshell quality and HHEP (*p* = 0.14) or HDEP (*p* = 0.05) (Table 1). Mortality of hens was significantly correlated with the eggshell quality (*p* = 0.03) with three breeders dying out of the 20 selected for poor eggshell quality and only one died from the good eggshell quality group. EW was not significantly different between good and poor eggshell groups (*p* = 0.33). The ratio of eggshell weight to total EW was significantly different between good and poor eggshell groups with good eggshells having a higher shell weight: EW ratio (*p* < 0.01) (Table 2). Shell weight per unit surface area (SWUSA) was significantly higher for eggs from the good eggshell group (*p* < 0.01). Shell Ca percentage of the total egg was significantly higher (*p* = 0.02) for eggs from the good eggshell group. SG was significantly higher for eggs from the good eggshell group (*p* < 0.01). Eggshell thickness was significantly thicker for eggs from the good eggshell group (*p* < 0.01).

**Table 1:** Production performance parameters from hens selected for eggshell quality from 30 to 46 weeks of age.^a^ ^,^ ^b^

	HHEP (%)	HDEP (%)	Mortality (%)	EW (g)
Eggshell quality^c^				
Good	52.1	54.1	5.0	64.8
Poor	53.8	60.3	15.0	63.0
*SEM*	2.0	5.2	5.0	2.5
*p value*	0.15	0.05	0.03	0.33

Abbreviations: EP, egg production; EW, egg weight; HDEP, hen‐d EP; HHEP, hen housed EP.

^a^ Values are presented as means ± SEM for the 16‐week production period.

^b^ *n* = 20 per treatment group.

^c^ Eggshell quality: Hens were defined as having good eggshell quality, having an average egg SG greater than 1.080, or having poor eggshell quality, having an average egg SG less than 1.080.

**Table 2:** Eggshell quality parameters from hens selected for eggshell quality from 30 to 46 weeks of age.^a^

	Shell:EW	SWUSA (mg/m^2^)	Shell calcium (%)	SG	Shell thickness (mm)
Eggshell quality^b^					
Good	0.093	80.56	41.69	2.028	0.42
Poor	0.076	65.25	33.12	1.064	0.33
*SEM*	0.032	21.4	10.42	0.054	0.025
*p value*	< 0.01	< 0.01	0.02	< 0.01	< 0.01

Abbreviations: EW, egg weight; SG, specific gravity; SWUSA, shell weight per unit surface area.

^a^ Values are presented as means ± SEM for the 16‐week production period.

^b^ Eggshell quality: Hens were defined as having good eggshell quality, having an average egg SG greater than 1.080, or having poor eggshell quality, having an average egg SG less than 1.080.

Bone alkaline phosphatase was significantly higher in good eggshell hens than poor eggshell hens (*p* = 0.02) (Table 3). Tartrate resistant acid phosphatase activity was higher in poor eggshell hens than good eggshell hens (*p* = 0.0022). Tibia ash percentage was significantly different between the bones of good and poor eggshell hens (*p* = 0.05) (Table 3). Tibia breaking strength was significantly higher in good eggshell hens compared to poor eggshell hens (*p* = 0.04). Bone alkaline phosphatase was significantly higher in the blood of the progeny hatched from eggs from good shell hens compared to the progeny of the poor eggshell hens (*p* < 0.01) (Table 4). Tartrate resistant acid phosphatase activity was significantly higher in the progeny hatched from eggs from poor eggshell hens compared to the progeny of the good shell hens (*p* = 0.0022). Body weight gain was not significantly different between the progeny of either good shell hens or poor shell hens (*p* = 0.56). Tibia ash was significantly higher in the progeny of the good eggshell hens compared to the progeny of the poor shell hens (*p* = 0.03). Tibia breaking strength was significantly higher from the progeny of the good eggshell hens compared to the progeny of the poor shell hens (*p* < 0.01).

**Table 3:** Blood & bone parameters from hens selected for eggshell quality from 30 to 46 weeks of age.^a^ ^,^ ^b^

	Bone alkaline phosphatase (pg/mL)	Tartrate resistant acid phosphatase (U)^c^	Tibia ash (%)	Tibia breaking strength (kg/mm)
Eggshell quality^d^				
Good	326.5	2203	58.0	7.26
Poor	253.2	4985	57.2	5.34
*SEM*	108.2	842.1	1.01	1.72
*p value*	0.02	0.002	0.05	0.04

^a^ Values are presented as means ± SEM for the 16‐week production period.

^b^ *n* = 20 per treatment group.

^c^ Activity of tartrate resistant acid phosphatase (TRAP) (U) was defined by the procedure by Lau et al. as the amount of non‐phytate phosphorus (NPP) cleaved in 1 h of incubation at 37°C.

^d^ Eggshell quality: Hens were defined as having good eggshell quality, having an average egg specific gravity (SG) greater than 1.080, or having poor eggshell quality, having an average egg SG less than 1.080.

**Table 4:** Blood, growth, & bone parameters from progeny of hens selected for eggshell quality at 2 weeks of age.^a^

	Bone alkaline phosphatase (pg/mL)	Tartrate resistant acid phosphatase (U)^b^	Body weight change (g)	Tibia ash (%)	Tibia breaking strength (kg/mm)
Eggshell quality^c^					
Good	372.3	18010	285.6	45.9	1.61
Poor	312.4	23590	285.4	42.2	1.47
*SEM*	30.4	1160	15.2	6.9	0.54
*p value*	< 0.01	0.002	0.56	0.03	0.03

^a^ *n* = 20 per treatment group.

^b^ Activity of tartrate resistant acid phosphatase (TRAP) (U) was defined by the described by Lau et al. (1987) as the amount of non‐phytate phosphorus (NPP) cleaved in 1 h of incubation at 37°C.

^c^ Eggshell quality: Progeny were created from hens which were defined as having good eggshell quality, having an average egg specific gravity (SG) greater than 1.080, or having poor eggshell quality, having an average egg SG less than 1.080.

**Figure 1:**
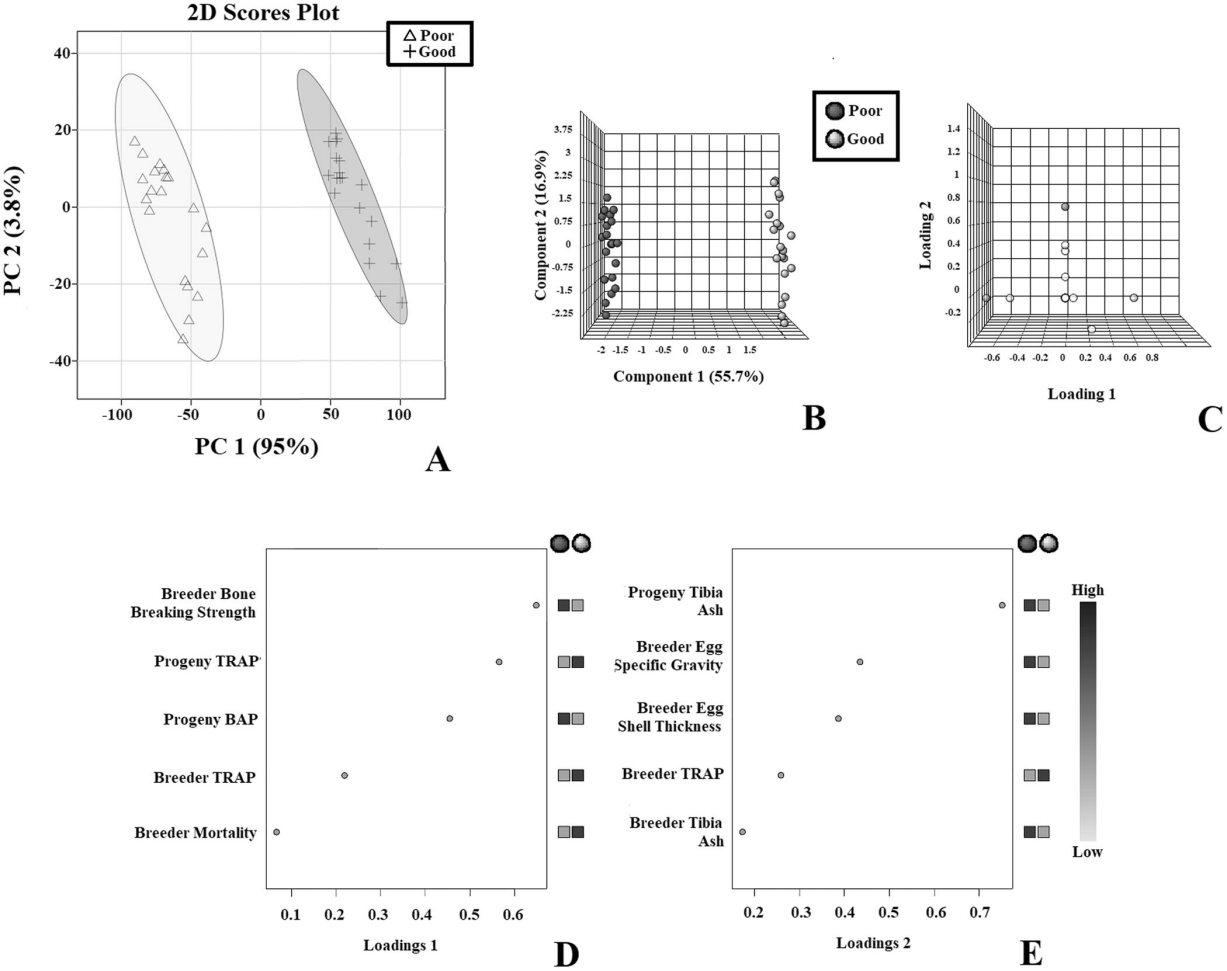
Distribution of phosphorus metabolism markers and discriminant analysis of bone strength and eggshell quality indicators for breeders and progeny. (A) 2D representation of principal component analysis (*n* = 30) of phosphorus metabolism indicators for breeders indicated as having poor or good eggshell quality. (B) 3D representation of component separation for sPLSDA (*n* = 30) of phosphorus metabolism indicators for breeders indicated as having poor or good eggshell quality. (C) 3D representation of loadings for sPLSDA (*n* = 30) of phosphorus metabolism indicators for breeders indicated as having poor or good eggshell quality. (D) Phosphorus metabolism indicators underlying separation of component 1 of the sPLSDA plot. (E) Phosphorus metabolism indicators underlying separation of component 2 of the sPLSDA plot. BAP, bone alkaline phosphatase; SWUSA, shell weight per unit surface area; TRAP, tartrate resistant acid phosphatase.

**Figure 2:**
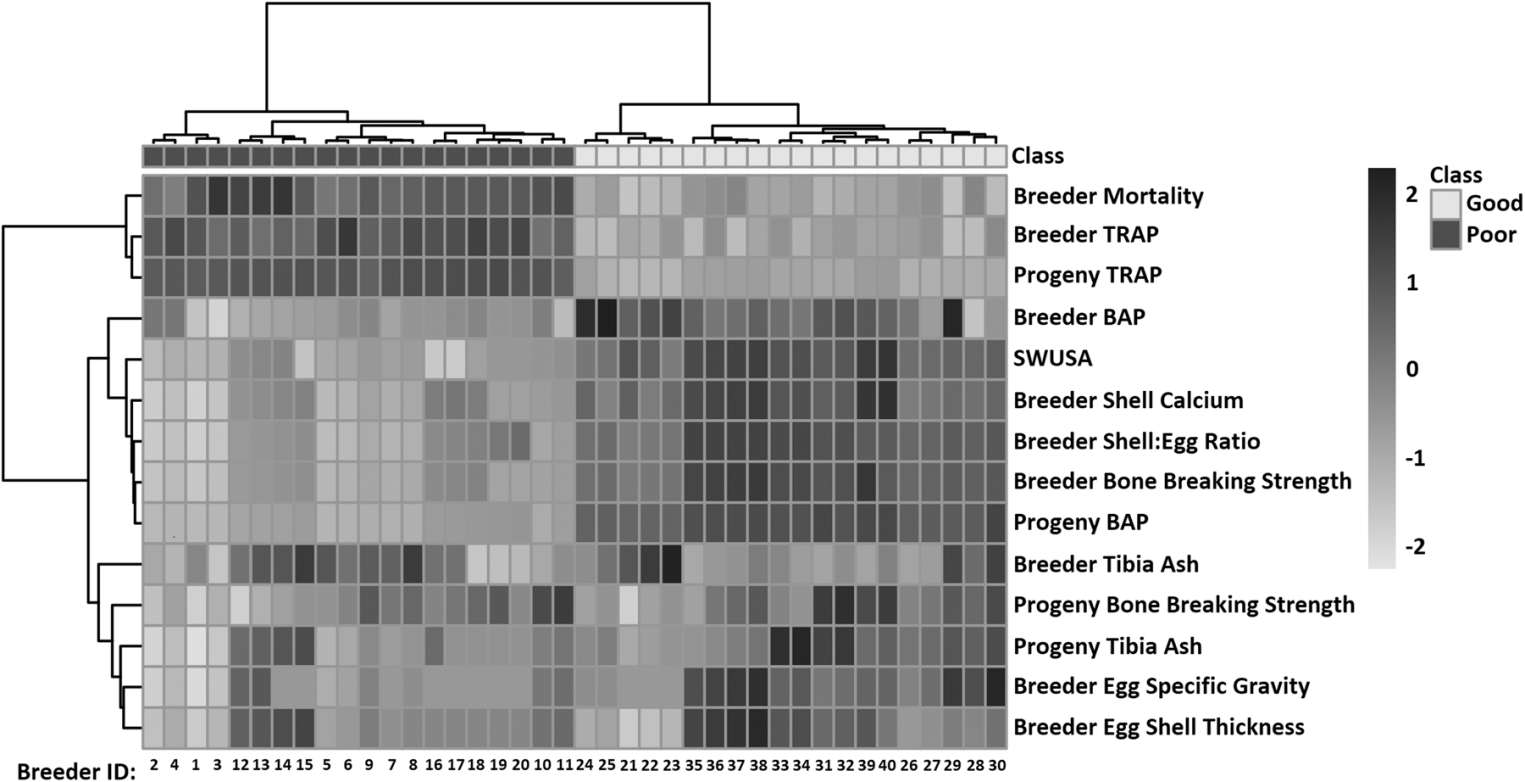
Hierarchical clustering Pearson's correlation heatmap plot of breeder hens and their progeny categorized into either poor or good shell quality groups and associated indicators of P metabolism. BAP, bone alkaline phosphatase; SWUSA, shell weight per unit surface area; TRAP, tartrate resistant acid phosphatase.

**Figure 3:**
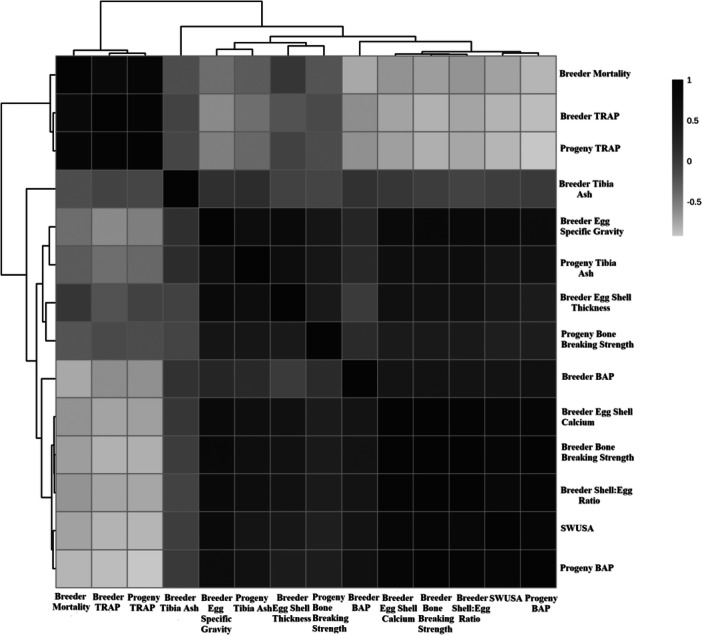
Pearson's correlation heatmap plot of plasma and bone P metabolism parameters categorised into either poor or good shell quality groups. BAP, bone alkaline phosphatase; SWUSA, shell weight per unit surface area; TRAP, tartrate resistant acid phosphatase.

sPLSDA was used to assess differences in phosphorus metabolism between the good and poor shell groups (Figure 1). The sPLSDA demonstrated clear separation of hen groups determined by eggshell quality **(**Figure 1A). Component 1‐based separation was mainly due to breeder bone strength, mortality, BAP, and TRAP concentrations including progeny (Figure 1B–D). Component 2‐based separations were due mainly to changes in breeder and progeny tibia ash, and breeder eggshell thickness and SG (Figure 1E). Correlation analysis provides further separation of the poor and good eggshell quality groups with good egg quality hens having a lower mortality, lower TRAP, and a lower related progeny TRAP. Conversely the poor eggshell quality group had lower BAP, shell:egg ratio, shell calcium, SWUSA, breaking strength, progeny BAP, egg SG, and eggshell thickness (Figure 2). Heatmapping analysis provides additional contrast of parameters measured against egg quality groups with BAP in breeders and progeny being positively correlated with stronger bones and eggshell, and TRAP being negatively correlated with said parameters (Figure 3).

## 4 Discussion

Bone problems in broilers and breeders including tibial dyschondroplasia and osteoporosis present challenges for producers (Jahejo and Tian 2021). Finding translatable calcium or phosphorus metabolism related biomarkers which can link parent stock with progeny would allow producers to select genetic lines which don't have these issues. The data presented from this work demonstrates that eggshell quality was correlated with breeder plasma BAP and TRAP. Additionally, the bone strength and health of progeny correlated with both eggshell quality and plasma BAP and TRAP of parent stock.

### 4.1 Eggshell Quality Correlation With BAP & TRAP

The mechanistic action of TRAP and bone resorption is thoroughly characterised in murine and avian models, however, there is limited application with broiler breeders (Dacke et al. 1993; Oddie et al. 2000). Ekmay et al. (2012) reported that breeder plasma concentration of TRAP is linked with eggshell synthesis and relative oviposition with distinct diurnal fluctuations signifying bone resorption and rebuilding. Notably TRAP has been utilized extensively in laying hens as a negatively correlated biomarker for bone metabolism and egg quality with its concentration being affected by age, nutrition, and genetics (Teng et al. 2020; Wei et al. 2021a, 2021b). Breeders in the good eggshell group in the current study had lower mortality, heavier EW, thicker shells, stronger bones, and had a higher Ca deposition in eggs relative to breeders in the poor eggshell group (Tables 1 and 2). These findings were substantiated by the difference in plasma activity of TRAP; breeders in the good eggshell quality group had 44% of the TRAP activity compared to TRAP activity for hens producing eggs with poor eggshell quality (Table 3). The pattern of eggshell quality and relation to TRAP is further supported by breeders in the good eggshell group having stronger bones with higher concentrations of tibia ash and breaking strength compared to the poor eggshell group (Table 3).

Like TRAP, the relationship between BAP and both egg quality and bone health has limited research in breeder hens (Al‐Daraji and H. M. Amen 2011). Previous work with layers has found that plasma BAP concentration is genetically linked and is positively correlated with EP and bone thickness, but not eggshell thickness (Wilcox, Van Vleck, and Shaffner 1962). The previous layer research disagrees with current results where there was no correlation between BAP and overall EP, and a positive association with BAP and eggshell thickness. The disparity between plasma BAP activity and egg traits may be due to the difference in genetics between layers and breeders as well as the gap in time with genetic selection changing animal metabolism (Tixier‐Boichard et al. 2012). Additionally, the methodology for measuring BAP was not the same across studies with previous research utilizing an indirect spectrophotometric assay whereas a direct quantitation with ELISA was utilized in present research study.

### 4.2 Progeny Bone Quality Correlation With TRAP & BAP

Bone metabolism in broilers has been extensively investigated due to the increasing incidence of tibial dyschondroplasia among flocks and associated cost to producers. Plasma TRAP is established to be an indicator of osteoclastic activity, overall osteocyte density, and is negatively associated with bone strength which can be modulated by many factors including diet concentrations of Ca, P and vitamin D, age of parent stock, egg size, growth rate, and genetic line (Yair et al. 2017; Zhang et al. 2022). The present work demonstrates that good shell quality hens had lower plasma concentrations of TRAP and created progeny which had lower levels of plasma TRAP with higher bone breaking strength and higher mineral ash content (Table 4). Comparatively, good shell quality progeny had 76% TRAP activity relative to poor shell quality progeny, similar to plasma concentrations from respective parent stock. These results agree with previous work in broilers which found TRAP and BAP to positively and negatively correlate, respectively, with femoral head necrosis and lower mineral density (Pang et al. 2021).

Plasma concentrations of BAP are indicative of osteoblast activity which promotes hydroxyapatite formation through synthesis of γ‐carboxyglutamate residues of type I collagen and is positively correlated with bone strength and bone mineral density in broilers (van Straalen et al. 1991). Plasma BAP concentrations are inversely influenced by the same parameters as TRAP, however, BAP has had less utilization in broiler research. Present data demonstrate that good shell quality progeny had 120% BAP activity relative to poor shell quality progeny with positive associations with bone mineral ash and health parameters. While the trend between plasma BAP and bone health is clear, one consideration is that previous work measuring broiler plasma BAP activity utilized spectrophotometric assays whereas the present study utilized a direct ELISA (de Souza Nakagi et al. 2013; Roberson and Edwards 1994).

### 4.3 Link Between Mineral Metabolism in Breeders & Progeny

Finding a heritable genetic link in breeder stock to improve broiler performance has been evaluated through various parameters from broiler mineral metabolism to osteocyte formation (Calini and Sirri 2007; Tompkins et al. 2022). Breeder age, genetic line, health, and dietary balance of Ca and P can influence progeny mineral metabolism through providing adequate nutrition for embryonic development which can modulate epigenetics (Yair, Uni, and Shahar 2012). It is well established that the dietary status of breeders directly influences eggshell quality, but genetics also influences eggshell thickness as breeders on the same diet can produce shells of varying thickness. The current study is based on the variability of eggshell quality by separating breeders fed the same diet into the two distinct eggshell groups which demonstrate the heritability of mineral metabolism in both groups’ progeny (Figure 1).

Dietary research with breeders which changed eggshell quality had no carryover to progeny with respect to bone health and performance, suggesting breeder genetics is the major determinant of broiler bone growth (Ekmay et al. 2012). Furthermore, the initial egg size and genetic line rate of growth both dictate overall broiler bone health with TRAP being negatively correlated with bone mineralisation (Yair et al. 2017). Notably previous research has demonstrated that breeder age does not influence progeny BAP and TRAP, but older breeders produced heavier chicks with wider tibias (Alfonso‐Torres et al. 2009). The present work demonstrates that parent stock selected for eggshell quality and eggshell density have stronger correlations with progeny bone health outcomes and plasma TRAP and BAP (Figures 2 and 3). The present work suggests bone metabolism heritability can be evaluated through eggshell quality, but not egg weight.

In conclusion, findings in the present study suggest there is a strong link between concentrations of TRAP, BAP, eggshell quality, and bone strength which are heritable from parent stock to progeny. Further research is necessary to determine how bone metabolism in broilers selected based upon breeder eggshell quality continues beyond day 14 and ultimately to market weight.

## Ethics Statement

Institutional Animal Care and Use Committee of University of Arkansas approved the study protocol (#13002) for welfare guidelines and husbandry practices performed in the experimental period. The committee is in strict compliance with Public Health Service Policy on Humane Care and Use of Laboratory Animals (PHS Policy), the USDA Animal Welfare Act and Regulations (AWAR), the institutional Animal Welfare Assurance and the University Policy on Animal Care and Use. The authors confirm that the ethical policies of the journal, as noted on the journal's author guidelines page, have been adhered to and the appropriate ethical review committee approval has been received. The authors confirm that they have followed EU standards for the protection of animals used for scientific purposes [and feed legislation, if appropriate].

## Conflicts of Interest

The authors declare no conflicts of interest.

## Supporting information

Supporting information.

## Data Availability

The data that support the findings of this study are available from the corresponding author upon reasonable request.
