# Supplementary material for: Utilization of Bone Alkaline Phosphatase (BAP) and Tartrate Resistant Acid Phosphatase (TRAP) as Biomarkers of Eggshell Quality and Bone Metabolism in Broiler Breeders and Progeny
Source: J Anim Physiol Anim Nutr (Berl). 2024 Dec 1;109(2):601–9. doi: 10.1111/jpn.14075 (PMC11919804; doi:10.1111/jpn.14075)
Supplement: Supplementary file 1 — Supporting information. [file JPN-109-601-s001.docx]

**Supplemental Tables**

**Utilization of Bone Alkaline Phosphatase (BAP) and Tartrate Resistant Acid Phosphatase (TRAP) as Biomarkers of Eggshell Quality and Bone Metabolism in Broiler Breeders and Progeny**

A. D. Magnuson^1^, N. Boonsinchai^2^, J. Caldas^3^, J. England^1^, C. Coon^1*^

Metabolism and Nutrition

**Abbreviation Key:** BAP, bone alkaline phosphatase; DEXA, dual energy x-ray absorptiometry; EP, egg production; EW, egg weight; HDEP, hen-d EP %; HHEP, hen-housed EP %; SG, specific gravity; sPLSDA, sparse partial-least squares discriminant analysis; SWUSA, shell weight per unit surface area; TRAP, tartrate resistant acid phosphatase.

^1^Center of Excellence for Poultry Science, University of Arkansas, Fayetteville, AR 72701

^2^CP Group, CP Tower 14F, 313 Silom Road, Bangkok, Thailand

^3^Aviagen Incorporated, Huntsville, Alabama 35805

^*^To whom correspondence should be addressed: [ccoon@uark.edu](mailto:ccoon@uark.edu)

Phone: 479-575-4134; Fax: 479-575-8755

**Supplemental Table 1**. Composition (%) of Cobb Breeder Diet

| **Ingredients** | (%) |
| --- | --- |
| Corn | 67.97 |
| Soybean Meal | 20.70 |
| Wheat Middlings | 6.00 |
| Poultry Oil | 1.00 |
| Calcium Carbonate | 6.70 |
| Dicalcium Phosphate | 1.81 |
| Sodium Bicarbonate | 0.27 |
| l-Lysine-HCl | 0.18 |
| dl-Methionine | 0.09 |
| Vitamin premix^[1](https://www.sciencedirect.com/science/article/pii/S0032579119402034#tbl1fn1)^ | 0.06 |
| Mineral premix^[2](https://www.sciencedirect.com/science/article/pii/S0032579119402034#tbl1fn2)^ | 0.07 |
| **Nutrients** |  |
| Crude Protein (%) | 16.0 |
| AME, kcal/kg | 2,860 |
| Calcium, % | 3.00 |
| Available P, % | 0.45 |
| Sodium, % | 0.19 |
| Lysine, % digestible | 0.88 |
| Methionine, % digestible | 0.38 |
| TSAA, % digestible | 0.57 |
| Threonine, % digestible | 0.54 |
| Isoleucine, % digestible | 0.61 |
| Valine, % digestible | 0.68 |
| Tryptophan, % digestible | 0.17 |
| Arginine, % digestible | 0.97 |

^1^ The vitamin premix contained per kilogram of diet: [vitamin A](https://www.sciencedirect.com/topics/agricultural-and-biological-sciences/retinol) from retinyl acetate, 60,000 IU; vitamin D_3_, 2,167 IU; [vitamin E](https://www.sciencedirect.com/topics/agricultural-and-biological-sciences/vitamin-e) from dl-α-tocopherol acetate, 33 IU; vitamin B_12_, 0.01 mg; vitamin B_6_ from pyridoxine mononitrate, 3 mg; [niacin](https://www.sciencedirect.com/topics/agricultural-and-biological-sciences/niacin), 27 mg; d-pantothenic acid, 10 mg; menadione, 2 mg; [folic acid](https://www.sciencedirect.com/topics/agricultural-and-biological-sciences/folic-acid), 0.87 mg; [thiamine](https://www.sciencedirect.com/topics/agricultural-and-biological-sciences/thiamine) from thiamine mononitrate, 3 mg; ethoxyquin, 67 mg; d-biotin, 0.3 mg; and [riboflavin](https://www.sciencedirect.com/topics/agricultural-and-biological-sciences/riboflavin), 7 mg.

^2^ The mineral premix contained zinc as [zinc sulfate](https://www.sciencedirect.com/topics/agricultural-and-biological-sciences/zinc-sulfate), 11.02%; manganese as manganese sulfate, 8.82%; iron as ferrous sulfate monohydrate, 5.29%; copper as copper sulfate, 1.03%; iodine as ethylenediamine dihydroiodide, 2,600 mg/kg; and selenium as sodium selenite, 400 mg/kg.

**Supplemental Table 2**. Feed Allocation Program for Breeder Hens

| **Week** | **Standard Feed Amount (g/bird)** |
| --- | --- |
| 20–21 | 45 |
| 21–22 | 104 |
| 22–23 | 109 |
| 23–24 | 114 |
| 24–25 | 118 |
| 5% production | 119 |
| 13% production | 121 |
| 21% production | 123 |
| 29% production | 126 |
| 37% production | 130 |
| 45% production | 134 |
| 53% production | 139 |
| 61% to peak | 144 |

**Supplemental Table 3**. Nutrient Profile of Chick Starter Diet

| **Nutrient** |  |
| --- | --- |
| Ingredient (%) |  |
| Corn | 61.7 |
| Soybean meal | 26.8 |
| Wheat middlings | 7.71 |
| Dicalcium phosphate^1^ | 1.83 |
| Ground limestone | 0.69 |
| Termin-8^2^ | 0.30 |
| Sodium chloride | 0.29 |
| Poultry fat | 0.25 |
| L-Lysine HCl | 0.10 |
| Alimet-MHA (liquid)^3^ | 0.10 |
| Choline Cl (70%) | 0.09 |
| Mineral premix^4^ | 0.06 |
| Copper sulfate | 0.05 |
| Vitamin premix^5^ | 0.04 |
| Ethoxyquin | 0.01 |
| Nutrient |  |
| ME (kcal/kg) | 2870 |
| CP | 19.00 |
| Crude fat | 2.82 |
| Lysine | 1.09 |
| Methionine+Cysteine | 0.76 |
| Calcium | 0.95 |
| Total phosphorus | 0.74 |
| Available phosphorus | 0.45 |

^1^18.5%, PCS Sales (SA) Inc., Northbrook, IL.

^2^ Mold inhibitor (Anitox Corp. Inc., Lawrenceville, GA).

^3^Alimet‐methionine hydroxy analog (Novus Int., St. Charles, MO).

^4^Mineral mix provided per kilogram of complete diet: Cu, 18 mg; I, 1.1 mg; Fe, 80 mg; Mn, 150 mg; Zn, 125 mg; and Se, 0.25 mg.

^5^Vitamin mix provided per kilogram of complete diet: [vitamin A](https://www.sciencedirect.com/topics/agricultural-and-biological-sciences/retinol), 10,000 IU; vitamin D_3_, 3,000 IU; [vitamin E](https://www.sciencedirect.com/topics/agricultural-and-biological-sciences/vitamin-e), 100 IU; vitamin K_3_, 3 mg; vitamin B_12_, 0.03 mg; [riboflavin](https://www.sciencedirect.com/topics/agricultural-and-biological-sciences/riboflavin), 8 mg; [niacin](https://www.sciencedirect.com/topics/agricultural-and-biological-sciences/niacin), 60 mg; [pantothenic acid](https://www.sciencedirect.com/topics/agricultural-and-biological-sciences/pantothenic-acid), 18 mg; [folic acid](https://www.sciencedirect.com/topics/agricultural-and-biological-sciences/folic-acid), 1 mg; [pyridoxine](https://www.sciencedirect.com/topics/agricultural-and-biological-sciences/vitamin-b6) HCl, 6 mg; [thiamine](https://www.sciencedirect.com/topics/agricultural-and-biological-sciences/thiamine) HCl, 3 mg; and [biotin](https://www.sciencedirect.com/topics/agricultural-and-biological-sciences/biotin), 0.2 mg.
